# Propofol ameliorates renal ischemia/reperfusion injury by enhancing macrophage M2 polarization through PPARγ/STAT3 signaling

**DOI:** 10.18632/aging.203107

**Published:** 2021-06-10

**Authors:** Zhaohui Liu, Yanli Meng, Yu Miao, Lili Yu, Qianjie Wei, Yuqing Li, Bing Zhang, Qiannan Yu

**Affiliations:** 1 Department of Anesthesiology, Cangzhou Central Hospital, Cangzhou, Hebei, China; 2 Department of Gastroenterology, Cangzhou Central Hospital, Cangzhou, Hebei, China; 3 Department of Neurosurgery, Cangzhou Central Hospital, Cangzhou, Hebei, China; 4 Department of Anesthesiology, Botou Hospital, Botou, Cangzhou, Hebei, China

**Keywords:** renal ischemia/reperfusion injury, propofol, peritoneal macrophage, inflammatory response

## Abstract

Propofol (Pro) confers protection against renal ischemia/reperfusion (rI/R) injury through incompletely characterized mechanisms. Since Pro has shown net anti-inflammatory properties as part of its beneficial effects, we examined the potential role of Pro in the modulation of macrophage polarization status during both rI/R injury *in vivo* and exposure of cultured peritoneal macrophages (PMs) to hypoxia/reoxygenation (H/R). Rats were subjected to 45-min r/IR surgery or a sham procedure and administered PBS (vehicle) or Pro during the ischemia stage. Pro administration attenuated rI/R-induced kidney damage and renal TNF-α, IL-6, and CXCL-10 expression. Enhanced macrophage M2 polarization, evidenced by reduced iNOS and increased Arg1 and Mrc1 mRNA levels, was further detected after Pro treatment both in the kidney, after rI/R *in vivo*, and in H/R-treated PMs. Pro administration also repressed phosphorylated signal transducer and activator of transcription 1 (p-STAT1) and increased p-STAT3, p-STAT6, and peroxisome proliferator-activated receptor-γ (PPARγ) mRNA levels in H/R-exposed PMs. Importantly, siRNA-mediated PPARγ silencing repressed Pro-mediated STAT3 activation in PMs and restored proinflammatory cytokine levels and prevented macrophage M2 marker expression in both rI/R-treated rats and cultured PMs. These findings suggest that Pro confers renoprotection against rI/R by stimulating PPARγ/STAT3-dependent macrophage conversion to the M2 phenotype.

## INTRODUCTION

Renal ischemia/reperfusion (rI/R) injury remains a chief problem for patients undergoing partial kidney resection and kidney replacement therapy [1, 2]. The high metabolic and oxygen demand and the extensive microvasculature of the kidneys make these organs particularly susceptible to I/R complications, which may lead to acute kidney injury (AKI) and progression to chronic kidney disease (CKD) [3]. Moreover, besides compromising kidney integrity and function, r/IR may also trigger dysfunction in distant organs, including lungs, heart, brain, and liver [4]. During the reperfusion stage, activation of the vascular endothelium facilitates the adherence and infiltration of immune cells, including lymphocytes, neutrophils, macrophages, and dendritic cells. While some immune cells participate actively in tissue healing, the release of proinflammatory mediators such as TNF- α, IL-6, and IL-1 by activated macrophages, T cells, and neutrophils triggers an inflammatory response that contributes to tissue damage and dysfunction [5, 6].

Due to insufficient understanding of the cellular players and the molecular mechanisms involved, current therapeutic strategies are scarce and often show limited benefits. Whereas perioperative interventions, such as ischemic preconditioning, fluid management, and intermittent hemodialysis may reduce rI/R injury and AKI development in selected surgical patients, pharmacological approaches have so far yielded disappointing or mixed results [7, 8]. Therefore, numerous preclinical studies, including our own, continue to explore both the specific contribution of innate immunity and potential approaches to restrict the inflammatory response, attenuate oxidative damage, modulate autophagy, and stimulate angiogenesis in the setting of rI/R injury [9–13].

Propofol (Pro), an intravenous anesthetic, is generally used for the sedation of surgical patients [14, 15]. Remarkably, Pro administration has shown to attenuate organ I/R injury and improve surgical outcomes in clinical and preclinical settings [11, 16–18]. As our past studies attest, lessening the production of proinflammatory cytokines lies at the core of Pro’s cytoprotective effects against rI/R injury [11, 16]. Therefore, the potential modulatory actions of Pro on macrophage transition from a pro-inflammatory (M1) to an anti-inflammatory (M2) phenotype is of great interest [19]. In the present study we used an *in vivo* rI/R model and an *in vitro* macrophage hypoxia/reperfusion (H/R) model to evaluate the effects of Pro administration on kidney inflammation and macrophage polarization status, and investigated the molecular mechanisms intervening in these effects.

## RESULTS

### Pro postconditioning reduces rI/R injury in rats

**Figure 1:**
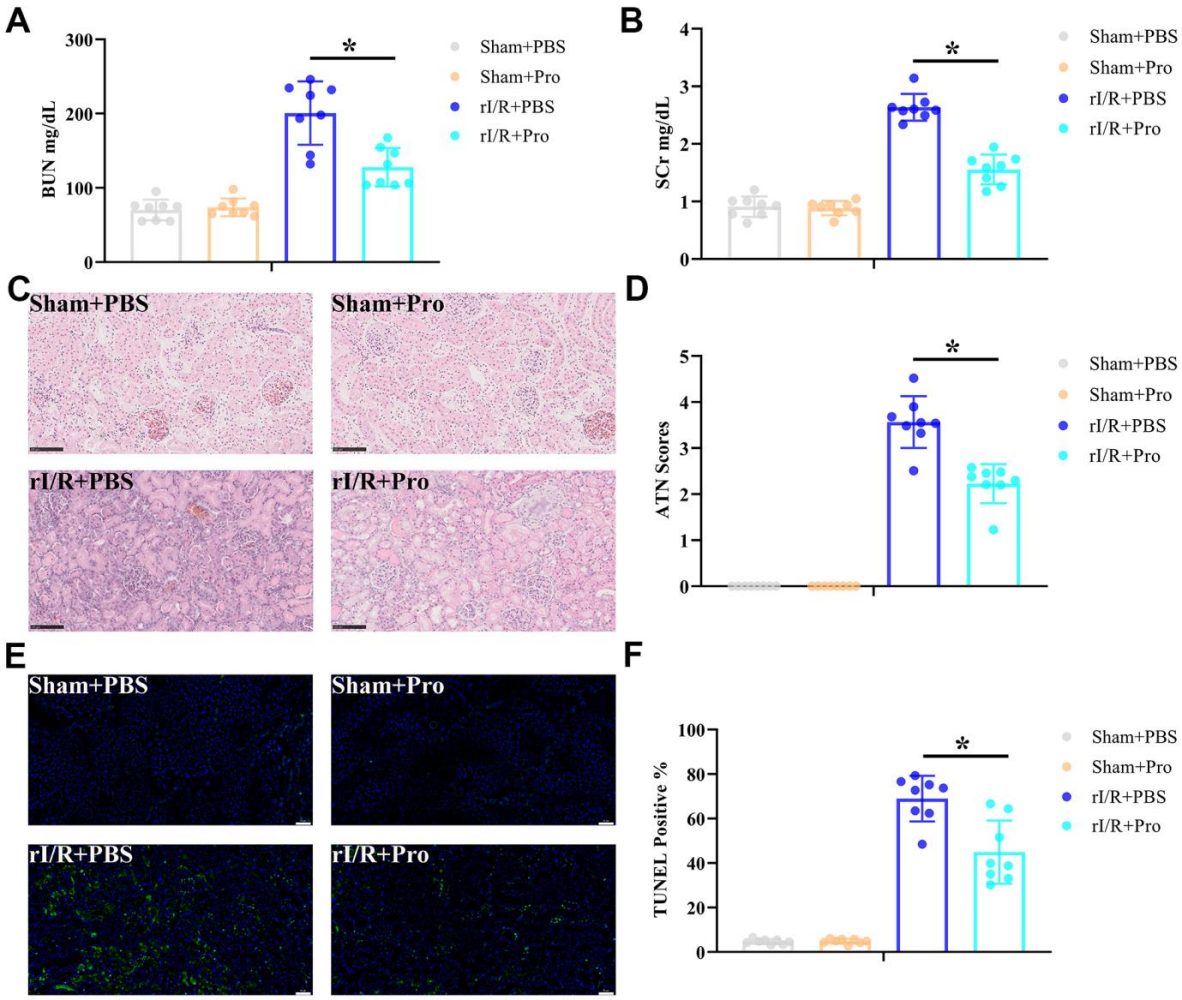
**Propofol attenuates kidney damage induced by rI/R injury.** Rats received rI/R or a sham procedure and Pro or vehicle (PBS) were administered via the femoral vein as described in Materials and Methods (n = 8 rats/group). Twenty-four hours after reperfusion, kidney injury was determined by assessing (**A**) serum BUN, (**B**) SCr, (**C**) kidney histopathology via H&E staining (200x magnification; scale bars = 100 μm), and (**D**) ATN scores. (**E**) Representative images of TUNEL staining of kidney sections (200x magnification; scale bars = 50 μm). DAPI was used for nuclear staining. (**F**) Quantification of TUNEL-positive cells in kidney sections. *P < 0.05 between the indicated groups.

To ascertain that Pro confers protection against rI/R injury *in vivo*, Sprague Dawley rats were subjected to rI/R or sham surgery and infused, at the onset of ischemia and over the reperfusion stage, with PBS (vehicle) or Pro as described in Materials and Methods. Rats were sacrificed 24 h later and serum obtained for blood urea nitrogen (BUN) and serum creatinine (SCr) determinations. Results showed that Pro administration significantly reduced serum BUN (Figure 1A) and SCr (Figure 1B) levels in rats exposed to rI/R. In turn, H&E staining showed that Pro administration attenuated renal injury (Figure 1C) and resulted in lower ATN scores (Figure 1D) compared to rI/R+PBS treatment. Moreover, compared to the latter group, TUNEL staining revealed a significant reduction in apoptosis in kidney samples from rI/R+Pro-treated animals (Figure 1E, 1F). No obvious changes in serum markers, kidney histology, or apoptosis were noted in sham-operated rats that received PBS or Pro. These findings confirmed that Pro administration significantly lessened classical manifestations of rI/R injury in rats.

### Pro administration attenuates rI/R-induced renal inflammation

**Figure 2:**
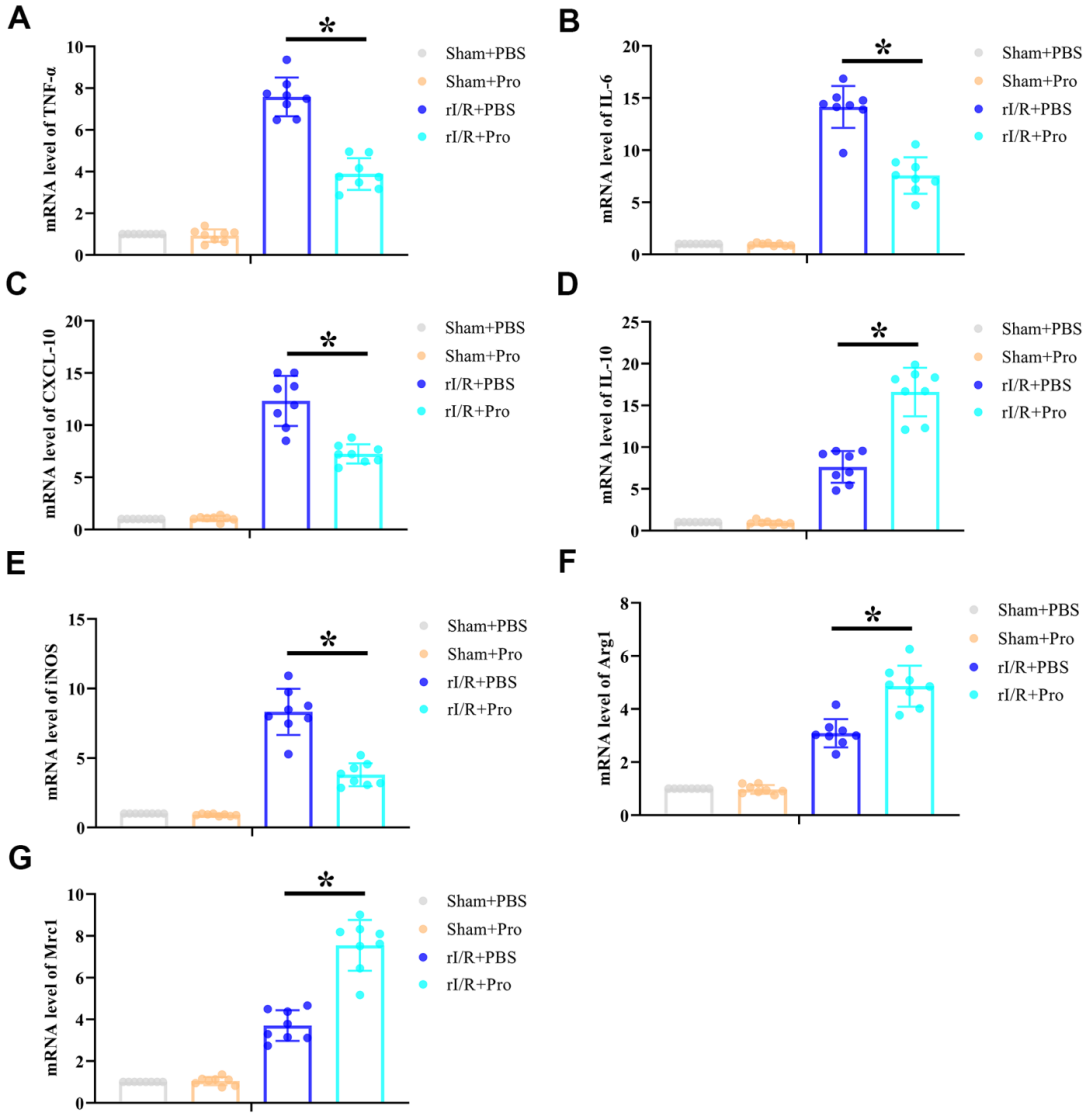
**Propofol postconditioning reduces renal proinflammatory cytokine levels and increases macrophage M2 polarization marker expression in rats subjected to rI/R.** Real-time PCR determination of relative renal mRNA expression levels of (**A**) TNF-α, (**B**) IL-6, (**C**) CXCL-10, (**D**) IL-10, (**E**) iNOS, (**F**) Arg1, and (**G**) Mrc1. *P < 0.05 between the indicated groups.

The inflammatory immune reaction is a major determinant of rI/R injury. To evaluate whether the protective effect of Pro on kidney function and structure after I/R is associated with a reduced inflammatory response, renal expression of proinflammatory and anti-inflammatory cytokines was quantified by real-time PCR. As shown in Figure 2A–2D, rI/R significantly activated the production of IL-6, TNF-α, CXCL-10, and IL-10 mRNA. Suggesting a net anti-inflammatory effect, Pro administration significantly repressed proinflammatory CXCL-10, IL-6, and TNF-α mRNA levels (Figure 2A–2C) but enhanced instead anti-inflammatory IL-10 mRNA production (Figure 2D) in kidney samples from rI/R-challenged rats. Furthermore, Pro administration during rI/R reduced renal expression of iNOS, a marker of M1 macrophages (Figure 2E) and enhanced the expression of macrophage M2 markers Arg1 and Mrc1 (Figure 2F, 2G). In contrast, no significant changes in cytokine or macrophage marker expression were observed in sham-operated, control rats administered PBS or Pro. These results suggest that Pro protects the kidneys from rI/R injury by alleviating the inflammatory response and promoting M1-to-M2 transition in macrophages.

### Pro treatment enhances M2 polarization in rat macrophages *in vitro*

**Figure 3:**
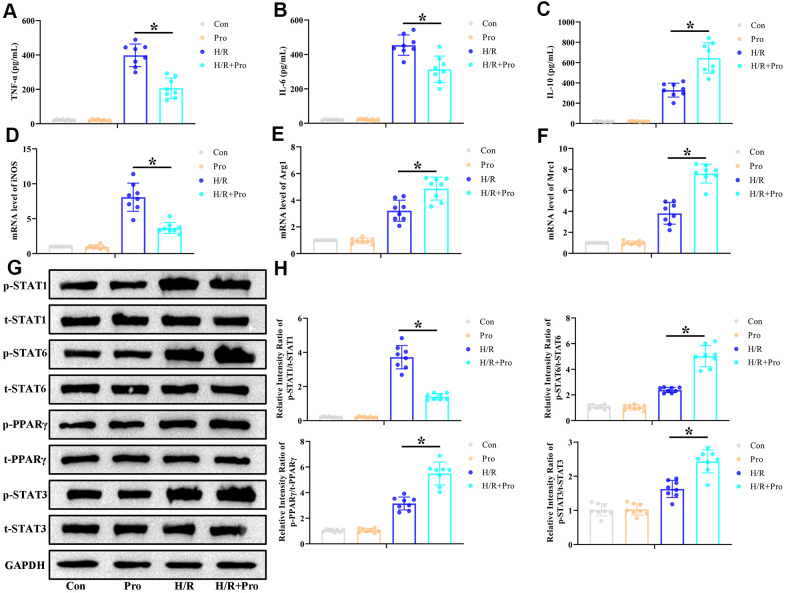
**Propofol promotes M2 polarization of macrophages *in vitro*.** PMs incubated with Pro or vehicle were kept and under normoxia or exposed to 6-h hypoxia followed by 24-h reoxygenation (H/R). (**A**–**C**) ELISA measurements of TNF-α, IL-6, and IL-10 levels in culture supernatants. (**D**–**F**) Real-time PCR assessment of iNOS, Arg1, and Mrc1 mRNA expression in cell lysates. (**G**, **H**) Representative western blot images and corresponding densitometric analysis of total and phosphorylated STAT 1/3/6 and PPARγ expression in cell lysates. Data were compared to control (Con) and GAPDH was used as loading control. *Con*: Normoxia+PBS; *Pro*: Normoxia+Pro; *H/R*: H/R+PBS. *P < 0.05 between the indicated groups.

Based on the above findings, and to determine whether Pro exposure can modulate the classical (M1) and alternative (M2) activation states in macrophages, rat peritoneal macrophages (PMs) were isolated and incubated with Pro or vehicle (PBS) during hypoxia/reoxygenation (H/R) stimulation. Cytokine expression and M1/M2 polarization markers were evaluated by ELISA and real-time PCR, respectively. In line with our *in vivo* findings, H/R strongly induced the expression of IL-6, TNF-α, and IL-10 in the supernatant of cultured PMs. However, lower concentrations of proinflammatory IL-6 and TNF-α (Figure 3A, 3B) and higher concentrations of anti-inflammatory IL-10 (Figure 3C) were detected for H/R+Pro PMs compared to control (H/R only) cells. Meanwhile, whereas H/R exposure stimulated the expression of markers of both M1 (iNOS) and M2 (Arg1 and Mrc1) phenotypes, Pro treatment reduced iNOS expression (Figure 3D) and increased the production of Arg1 and Mrc1 mRNA (Figure 3E, 3F).

The critical influence of STAT3 and PPARγ activation in modulating macrophage M1/M2 phenotype conversion has been described in previous studies [20, 21]. Therefore, we conducted western blot assays to assess whether STAT3 and PPARγ expression in PMs is affected by exposure to Pro. We found that H/R increased p-STAT1, p-STAT3, p-STAT6, and p-PPARγ expression, whereas Pro incubation reduced p-STAT1 and increased p-STAT3, p-STAT6, and p-PPARγ levels in H/R-treated PMs (Figure 3G, 3H). Once again, in control, non-H/R-treated cells, Pro alone did not alter the expression of these proteins. These findings indicate that Pro activates STAT3 and PPARγ signaling in PMs exposed to H/R.

### Pro promotes M2 polarization of H/R-challenged macrophages through the PPARγ/STAT3 pathway

**Figure 4:**
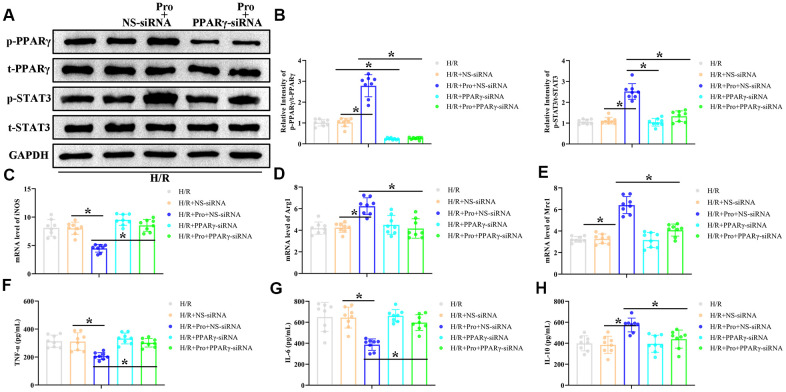
**Propofol enhances M2 polarization of macrophages *in vitro* through the PPARγ/ STAT3 pathway.** PMs were transfected with PPARγ-siRNA or a non-silencing control siRNA (NS-siRNA) and preincubated with Pro or vehicle before H/R treatment. (**A**, **B**) Representative western blot images and corresponding densitometric analysis of total and phosphorylated PPARγ and STAT 3 expression in cell lysates. Data were compared to control (Con) and GAPDH was used as loading control. (**C**–**E**) Real-time PCR analysis of relative iNOS, Arg1, and Mrc1 mRNA levels in cell lysates. Data were normalized against corresponding measurements in non-transfected, non-treated macrophages. (**F**–**H**) ELISA measurements of TNF-α, IL-6, and IL-10 contents in culture supernatants. *P < 0.05 between the indicated groups.

To investigate whether PPARγ/STAT3 signaling mediates the switch to the M2 phenotype induced by Pro in H/R-treated PMs, PPARγ expression was suppressed in these cells by transfection with PPARγ-directed siRNA. As indicated in Figure 4A, 4B, PPARγ siRNA efficiently repressed PPARγ activation in both control and Pro-treated cells and blocked STAT3 activation in Pro-treated PMs. Notably, PPARγ silencing also abrogated the effects of Pro on iNOS, Arg1, and Mrc1 expression, but did not affect their levels in PMs cultured without Pro (Figure 4C–4E). Also importantly, PPARγ depletion abrogated the reduction in pro-inflammatory IL-6 and TNF-α levels and the increase in anti-inflammatory IL-10 mRNA production induced by Pro (Figure 4F–4H). These findings suggest that Pro promotes polarization of H/R-challenged macrophages towards the M2 phenotype by enhancing PPARγ-mediated activation of STAT3.

### PPARγ activation mediates the renoprotective effect of Pro against rI/R injury by promoting macrophage M2 polarization

**Figure 5:**
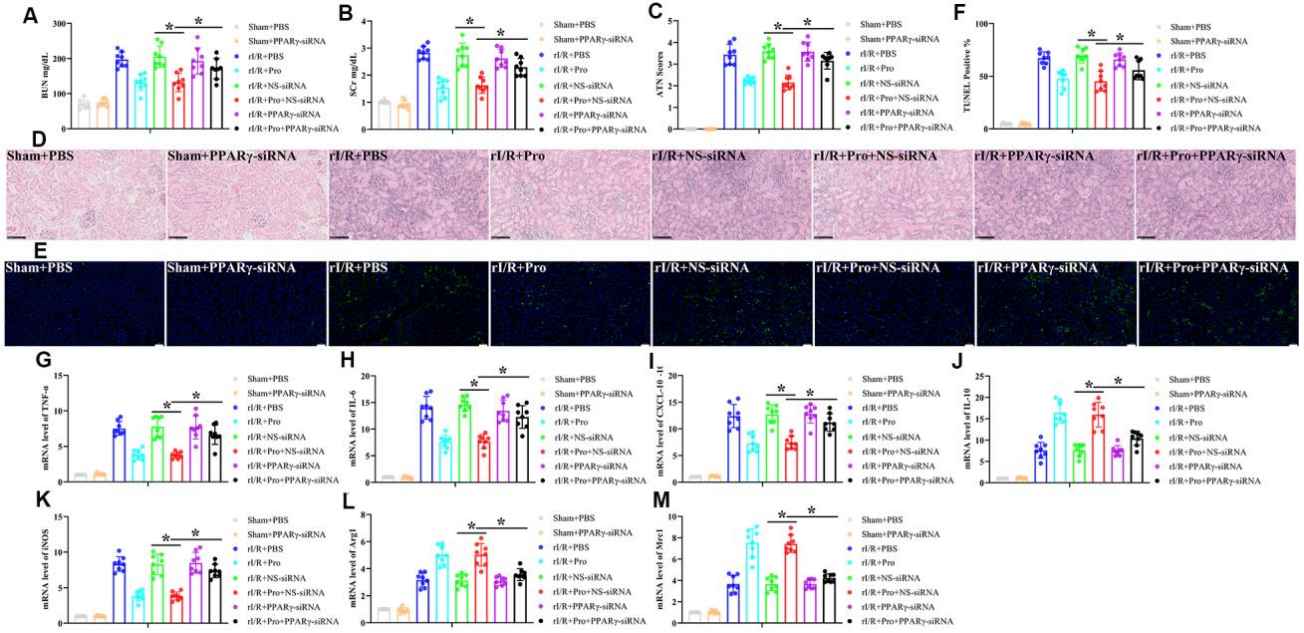
**PPARγ silencing inhibits Pro-mediated renoprotection and M2 polarization of kidney macrophages.** Rats were injected with PPARγ-siRNA or a negative control siRNA (NS-siRNA) before rI/R or sham surgery (n = 8/group). Twenty-four hours after reperfusion, kidney injury was determined by assessing (**A**) serum BUN, (**B**) SCr, (**C**) ATN scores, and (**D**) kidney histopathology via H&E staining (200x magnification; scale bars = 100 μM). (**E**) Representative images of TUNEL staining of kidney tissues (200x magnification; scale bars = 50 μM). DAPI was used for nuclear staining. (**F**) Quantification of TUNEL-positive cells in kidney sections. (**G**–**J**) Real-time PCR analysis of relative TNF-α, IL-6, CXCL-10, and IL-10 expression levels in kidney samples. (**K**–**M**) Real-time PCR analysis of relative iNOS, Arg1, and Mrc1 mRNA expression levels in kidney samples. *P < 0.05 between the indicated groups.

Finally, and to determine whether PPARγ activation is involved in the renoprotective actions of Pro against rI/R, we silenced PPARγ *in vivo* by administering a tail vein injection of a PPARγ siRNA premixed with mannose-conjugated polymers 4 h before rI/R surgery. Analyses carried out 24 h post-reperfusion indicated that PPARγ-siRNA treatment had no effect on sham rats but attenuated or abolished the renoprotective actions of Pro on rI/R-treated rats. This was evidenced by higher serum BUN and SCr concentrations, higher ATN scores for tissue samples, and increased numbers of TUNEL-positive cells compared to both rI/R+Pro and rI/R+Pro+NS-siRNA treatment groups (Figure 5A–5F). In addition, compared to the last two groups, PPARγ silencing significantly increased TNF-α, IL-6, and CXCL-10 mRNA levels and reduced IL-10 mRNA production in kidney samples from rats subjected to rI/R (Figure 5G–5J). Suggesting a critical role for Pro-mediated PPARγ activation in the conversion of renal macrophages to the M2 phenotype, PPARγ depletion in rI/R-treated rats prevented the decrease in iNOS and the increase in Arg1 and Mrc1 mRNA levels induced by Pro administration (Figure 5K–5M).

## DISCUSSION

Proinflammatory innate immune activation exerts a crucial role in driving the full progression of inflammatory kidney damage [22]. The latter is a key pathogenic event in rI/R injury and is largely mediated by the release, both locally and into the circulation, of endogenous ligands termed damage-associated molecular patterns (DAMPs) [23]. Since DAMPs act mainly on dendritic cells and macrophages through pattern recognition receptors (PRRs) to activate the release of proinflammatory factors, therapeutic strategies aimed at modulating innate immune activation seem vital to lessen kidney damage mediated by I/R [22, 24]. Extensive evidence indicates that conversion of tissue-infiltrating macrophages from a proinflammatory M1 phenotype to an anti-inflammatory M2 phenotype accelerates healing and promotes tissue neoangiogenesis following acute trauma and I/R injury [25]. To the best of our knowledge, this is the first study to show that Pro administration following acute kidney I/R injury induces the expression of macrophage M2 polarization markers in a PPARγ/STAT3-dependent manner.

Many biological and nonbiological treatment approaches have been described to protect the kidney from I/R injury [26–28]. Among those, ischemic preconditioning is one of the most studied ones [27]. In turn, a number of natural compounds demonstrated renoprotective effects in the setting of rI/R by regulating the activity of proteases, reducing oxidative stress, stimulating angiogenesis, and hampering the inflammatory response [28, 29]. Recently, a number of innovative approaches based on administration of fetal kidney cells [30] and gene- and RNAi-based therapies [31, 32] have shown also promising preclinical results.

The renoprotective actions of pre- or post-ischemia Pro administration against rI/R injury have been substantiated by several preclinical studies. In rat models, mitigation of rI/R injury mediated by Pro has been associated with antioxidant and anti-inflammatory effects related to induction of HO-1 expression [33], decreased MDA production [34], reduced expression of immune cell-derived mediators of inflammation and oxidative stress such as myeloperoxidase, TNF-α, and IL-1β [35], and prevention of tubular apoptosis [24]. Details on the molecular mechanisms underlying Pro-mediated renoprotection were provided by Wei et al., who showed that Pro preconditioning reduced kidney injury by inhibiting the PI3K/AKT/mTOR axis in a mouse model of rI/R [16], and by Su et al., who reported that Pro preconditioning lessened rI/R injury in rats by preventing endoplasmic reticulum stress [36]. In turn, our preceding research showed that Pro post-conditioning attenuated rI/R-induced acute lung injury by inhibiting autophagy and apoptosis in rat lung cells [11].

While the above studies highlighted several mechanisms by which Pro mediates renal protection following rI/R, its specific effects on macrophage activation status remain uncertain. Thus, based on available evidence supporting the anti-inflammatory actions of Pro in the reperfused kidney, we tested the hypothesis that prominent macrophage polarization towards the M2 phenotype contributes to Pro-mediated renoprotection. Through M1/M2 marker expression assays, we obtained robust, direct evidence *in vitro* and indirect evidence *in vivo* that macrophage M1-to-M2 transition takes place in the reperfused rat kidney after infusion of Pro. More importantly, a significant insight into the renoprotective mechanisms of Pro was herein provided by demonstrating a critical connection between PPARγ activation and macrophage M2 conversion during the reperfusion stage.

PPARγ, a member of the PPAR subfamily of nuclear receptors, is broadly expressed in several tissues, including the kidney [37, 38]. The anti-inflammatory activity of PPARγ in cells of the myeloid lineage has been described as crucial in governing proinflammatory cytokine production, myeloid-derived suppressor cell (MDSC) expansion, immunosuppression, and tumor progression [39, 40]. Significant roles of PPARγ have also been discovered in organ I/R injury [41–43], and some studies have described the impact of PPARγ signaling in the modulation of macrophage polarization status [20, 44]. In line with such evidence, our study showed that Pro exposure enhanced PPARγ activation and promoted a switch towards an M2 activation state. This was evidenced by reduced iNOS and enhanced Mrc1 and Arg1 mRNA production both directly, through *in vitro* assays, but also indirectly, through expression analyses of kidney tissue *ex vivo*. In both instances, PPARγ depletion enhanced M1 and prevented M2 macrophage marker expression and abrogated the reduction in proinflammatory cytokine production promoted by Pro.

Studies on the modulatory activity of Pro on macrophage polarization are limited. Kochiyama et al. reported that Pro suppressed production of IL-6 and IL-1β but did not alter TNF-α production during M1 polarization of human macrophages. In contrast, mRNA levels of M2 markers such as IL-10, TGF-β, and CD206 were not impacted by Pro during M2 polarization [19]. Although species-specific differences need to be considered, these findings and ours strongly suggest that Pro attenuates kidney damage due to rI/R at least in part by halting M1 polarization and stimulating the M2 phenotype in mammalian macrophages.

In conclusion, our research suggested that Pro postconditioning reduces kidney damage resulting from rI/R injury by enhancing M2 polarization of macrophages in a PPARγ/STAT3/-reliant manner. Limitations of our study include mainly lack of direct proof of Pro-induced M2 marker expression in macrophages *in situ* and demonstration of a direct modulatory role of STAT3 in this transition [45]. Nevertheless, our results suggest that strategies to activate PPARγ signaling in target tissues might be effective to ameliorate organ damage and dysfunction resulting from I/R.

## MATERIALS AND METHODS

### Renal ischemia/reperfusion model

Eight-week-old male Sprague-Dawley rats were acquired from Shanghai Lingchang Biotech Co., Shanghai, China. Rats were kept under specific pathogen-free conditions and were provided with standard chow and sterile acidified water *ad libitum*. All rats received humane care as stated by the protocol approved by the Institutional Animal Care and Use Committee of Cangzhou Central Hospital. A rat model of rI/R injury was generated as described previously [46]. Rats were intraperitoneally sedated with chloral hydrate (10%, 0.3 g/kg) and bilateral renal ischemia was completed by clamping the renal pedicles with microvascular clamps. After 45 min of ischemia, the clip was removed to initiate kidney reperfusion. The rats were kept in a warm container (24° C-29° C) and sacrificed 24 h later. Sham rats underwent an identical procedure but without vascular blocking. At the onset of reperfusion, a bolus of Pro (10 mg/kg; dissolved in PBS) or an identical volume of PBS was infused continuously for 45 min through the left femoral vein, followed by infusion of Pro (5 mg/kg/h) or PBS for an additional 23 h [11]. Four experimental groups (n = 8 rats/group) were thus defined: 1) Sham+PBS (sham surgery plus PBS infusion); 2) Sham+Pro (sham surgery plus Pro infusion); 3) rI/R+PBS (rI/R surgery plus PBS infusion); and 4) rI/R+Pro (rI/R surgery plus Pro infusion). To minimize pain, all rats were pre-operatively treated with carprofen (6 mg/kg) via intraperitoneal injection.

### Serum biochemistry and kidney histopathology

After 24 h of reperfusion, rats were sacrificed and blood and kidney sections were obtained. An AU5400 automated biological analyzer (Olympus, Tokyo, Japan) was used to determine blood urea nitrogen (BUN) and serum creatinine (SCr) concentrations. Hematoxylin and eosin (H&E) were used to stain 4-μm paraffin-embedded kidney tissue sections. Acute tubular necrosis (ATN) standards were used to classify the severity of rI/R.

### TUNEL analysis

Apoptosis analysis of kidney sections was performed using a TUNEL fluorescence detection kit (Beyotime, Shanghai, China) according to the manufacturer’s instructions.

### Peritoneal macrophage culture

Rat peritoneal macrophages (PMs) were isolated as described previously [47]. In brief, animals were intraperitoneally injected with ice-cold PBS and peritoneal fluid was aspirated and centrifuged at 300 g for 5 min. After discarding the supernatant, the cells were washed twice with RPMI-1640 medium containing 15% FBS, inoculated into culture dishes, and placed in an incubator at 37° C/5% CO_2_. After 2 h, nonadherent cells were washed out with PBS. The remaining cells (adherent PMs) were then replated and cultured overnight before downstream experiments. To simulate I/R *in vitro*, PMs were subjected to hypoxia/reoxygenation [H/R; hypoxia (1% O_2_, 94% N_2_, 5% CO_2_) for 6 h and reoxygenation for 24 h]. In some studies, Pro (100 μM) or an equal volume of PBS (control) was added to the culture medium simultaneously with the H/R stimulus.

### Quantitative real-time PCR

Total RNA was extracted from frozen kidney sections and cultured macrophage lysates and reverse-transcribed into cDNA using the SuperScript III System (Invitrogen, Carlsbad, CA, USA). SYBR Green Master Mix (Roche, Indianapolis, IN) was used to perform real-time PCR following a standard protocol. Primer sequences utilized in these assays were listed in Table 1.

**Table 1:** Primer sequences for qRT-PCR.

**Gene**	**Sequence**
Rat-TNF-α	F5′-GCATGATCCGAGATGTGGAACTGG-3′
	R5′-CGCCACGAGCAGGAATGAGAAG-3′
Rat-IL-6	F5′-AGGAGTGGCTAAGGACCAAGACC-3′
	R5′-TGCCGAGTAGACCTCATAGTGACC-3′
Rat-CXCL-10	F5′-GGGATCCCTCTCGCAAGAA-3′
	R5′-CTCAGCGTCTGTTCATGGAAGT-3′
Rat-IL-10	F5′-CCCAGAAATCAAGGAGCATTTG-3′
	R5′-CAGCTGTATCCAGAGGGTCTTCA-3′
Rat-iNOS	F5′-TGGGTGAAAGCGGTGTTCTT-3′
	R5′-TAGCGCTTCCGACTTCCTTG-3′
Rat-Arg1	F5′-CTACCTGCTGGGAAGGAAG-3′
	R5′-GTCCTGAAAGTAGCCCTGTC-3′
Rat-Mrc1	F5′-GGGGTTGTTGCTGTTGATGT-3′
	R5′-GCTCGAAACGGAAAAGGTTC-3′
Rat-GAPDH	F5′-GTCCATGCCATCACTGCCACTC-3′
	R5′-GATGACCTTGCCCACAGCCTTG-3′

### ELISA

IL-10, IL-6, and TNF-α concentrations in cell culture supernatants were determined using ELISA kits (Beyotime, Shanghai, China) according to the manufacturer’s procedures.

### Western blotting

PM cultures were lysed to obtain total protein extracts. Antibodies against total and phosphorylated signal transducer and activator of transcription proteins (t-STAT1/p-STAT1; t-STAT3/p-STAT3; t-STAT6/p-STAT6), total and phosphorylated peroxisome proliferator-activated receptor-γ (t-PPARγ/p-PPARγ), and GAPDH (all acquired from Beyotime Biotechnology, Shanghai, China) were used to perform western blotting following a standard protocol.

### siRNA-mediated PPARγ silencing

PPARγ siRNA (Beyotime, China) was used to deplete PPARγ mRNA levels. Cultured PMs were transfected with 50 nM PPARγ-targeted or non-silencing control siRNA (NS-siRNA) using Lipofectamine (Invitrogen, USA) as instructed by the manufacturer. To deplete PPARγ mRNA *in vivo*, 20 mg/kg PPARγ siRNA (or a negative control siRNA) premixed with mannose-conjugated polymers (Polyplus-Transfection, Strasbourg, France) was injected via the tail vein 4 h before the beginning of kidney ischemia.

### Statistical analysis

Results are presented as the mean ± S.D. Statistical analysis were performed with GraphPad Prism software using one-way analysis of variance (ANOVA) followed by Bonferroni’s post hoc test. *P* < 0.05 was considered significant.
